# Clinical Factors and Disease Course Related to Diagnostic Delay in Korean Crohn’s Disease Patients: Results from the CONNECT Study

**DOI:** 10.1371/journal.pone.0144390

**Published:** 2015-12-08

**Authors:** Chang Mo Moon, Sung-Ae Jung, Seong-Eun Kim, Hyun Joo Song, Yunho Jung, Byong Duk Ye, Jae Hee Cheon, You Sun Kim, Young-Ho Kim, Joo Sung Kim, Dong Soo Han, CONNECT study group

**Affiliations:** 1 Department of Internal Medicine, School of Medicine, Ewha Womans University, Seoul, Republic of Korea; 2 Department of Internal Medicine, Jeju National University School of Medicine, Jeju, Republic of Korea; 3 Department of Internal Medicine, Soonchunhyang University College of Medicine, Cheonan Hospital, Cheonan, Republic of Korea; 4 Department of Internal Medicine, University of Ulsan College of Medicine, Seoul, Republic of Korea; 5 Department of Internal Medicine, Yonsei University College of Medicine, Seoul, Republic of Korea; 6 Department of Internal Medicine, Inje University College of Medicine, Seoul Paik Hospital, Seoul, Republic of Korea; 7 Department of Internal Medicine, Sungkyunkwan University School of Medicine, Seoul, Republic of Korea; 8 Department of Internal Medicine, Seoul National University College of Medicine, Seoul, Republic of Korea; 9 Department of Internal Medicine, Hanyang University College of Medicine, Guri Hospital, Guri, Republic of Korea; INSERM, FRANCE

## Abstract

Diagnostic delay frequently occurs in Crohn’s disease (CD) patients because of diagnostic limitations. However, diagnostic delay and its related factors remain poorly defined. Therefore, we aimed to identify the predictors associated with diagnostic delay and to evaluate the impact of diagnostic delay on clinical course in a Korean CD patient cohort. We performed a multicenter retrospective analysis of 1,047 CD patients registered in the Crohn’s Disease Clinical Network and Cohort study in Korea. The mean interval of diagnostic delay was 16.0 ± 33.1 months. Multivariate analysis showed that older age at diagnosis (≥40 years) (*p* = 0.014), concomitant upper gastrointestinal (UGI) disease (*p* = 0.012) and penetrating disease behavior at diagnosis (*p* = 0.001) were positively associated with long diagnostic delay (≥18 months). During the longitudinal follow-up, long diagnostic delay was independently predictive of further development of intestinal stenosis (hazard ratio [HR], 1.43; 95% confidence interval [CI], 1.07–1.93; *p* = 0.017), internal fistulas (HR, 1.62; 95% CI, 1.12–2.33; *p* = 0.011), and perianal fistulas (HR, 1.38; 95% CI, 1.06–1.80; *p* = 0.016). However, as for the risk of abscess formation, bowel perforation, and CD-related abdominal surgery, no significant association with diagnostic delay was observed. Older age at diagnosis, UGI involvement, and penetrating behavior are associated with long diagnostic delay in Korean CD patients. Moreover, diagnostic delay is associated with an increased risk of CD-related complications such as intestinal stenosis, internal fistulas, and perianal fistulas.

## Introduction

Crohn’s disease (CD) is a chronic relapsing and destructive inflammatory condition of the gastrointestinal tract that progresses to bowel damage related to impaired functioning. When CD patients are followed over time, patients with inflammatory behavior tend to experience structural destruction of the bowel, such as stricture or internal fistulas [1,2]. Recently, studies have shown that early diagnosis and intensive treatment of CD are necessary to prevent bowel damage and unexpected surgery in patients [3–5]. In addition, studies reported that early intensive treatment with immunomodulators or biologics showed superior clinical outcomes to conventional treatment in newly diagnosed CD patients [3,4].

However, a long diagnostic delay occurs in a considerable number of CD patients [6,7] because of the unspecific symptoms of CD and unsatisfactory diagnostic accuracy of tests for CD. Considering that intestinal inflammation occurs after the onset of the first symptoms in CD patients, reducing diagnostic delay could have clinical implications in real clinical practice. Identification of factors associated with CD can allow early treatment and prevent an unfavorable course in CD patients. Several studies have evaluated clinical factors associated with diagnostic delay in Western patients [8,9] and few studies have examined these factors in Eastern patients [10]. Considering the significant differences in the epidemiologic and clinical features of CD according to ethnicities and environmental factors [11–13], diagnostic delay and its associated factors may differ with countries.

Therefore, the present study aimed to evaluate the clinical factors associated with a long diagnostic delay in Korean CD patients. In addition, we investigated the influence of diagnostic delay on the clinical course of CD, including the occurrence of complications, in our CD patient cohort.

## Materials and Methods

### Study subjects

This study analyzed clinical data obtained from 1,047 Korean CD patients diagnosed between January 1, 2000 to December 31, 2008. All data were retrieved from the Crohn’s Disease Clinical Network and Cohort (CONNECT) study, a multicenter retrospective cohort study of CD patients, which was conducted in 30 university hospitals and 2 local hospitals nationwide in Korea [14]. All patients were diagnosed and treated by inflammatory bowel disease (IBD) specialists who are members of the Korean Association for the Study of Intestinal Diseases. Diagnosis of CD was made on the basis of clinical, endoscopic, radiological, and histopathologic characteristics of the patients [15,16]. The following patients were excluded from this study: those who were diagnosed with or suspected to have intestinal tuberculosis, intestinal Behçet’s disease, or indeterminate colitis; those who were followed up for < 6 months; and those with incomplete medical records. Because this is retrospective observational study, written consent could not be obtained. But, all clinical data were analyzed anonymously to protect patient privacy. This study was approved by the Institutional Review Board of Ewha Womans University Mokdong Hospital (EUMC 2014-10-034) and conducted in accordance with the ethical guidelines of the Declaration of Helsinki.

### Data collection, definition and assessment of clinical outcomes

The demographic and clinical characteristics including age at first diagnosis, gender, family history of IBD, history of previous abdominal surgery, and follow-up duration were analyzed. Follow-up duration was defined as the period from the CD diagnosis to the latest follow-up. Disease location and behavior at initial presentation were classified according to the Montreal Classification [17]. Prescribed medications including 5-aminosalicylic acid, antibiotics, oral corticosteroids, azathioprine/6-mercaptopurine, methotrexate, and anti-tumor necrosis factor α (anti-TNFα) were also recorded. Data were obtained by reviewing the medical records of the CD patients at each institute. Diagnostic delay was defined as the time interval from the onset of the first symptoms to the establishment of the CD diagnosis. The first CD-related symptoms and their onset were evaluated in all patients by a review of the physician’s notes. Specific symptoms of CD included bloody diarrhea, chronic or recurrent diarrhea or abdominal pain accompanied by noticeable weight loss, general weakness, and fever. To investigate the association between clinical factors and diagnostic delay in CD patients, a time interval of ≥18 months was defined as a “long diagnostic delay.” Further, to evaluate the clinical effects of the diagnostic delay, the primary outcome was measured as the time to development of the following CD-related complications: intestinal stenosis, internal fistula, perianal fistula, abscess formation, bowel perforation, and disease-related abdominal surgery. Internal fistulas included all fistulas from the bowel to other organs, such as entero-enteric, enterocutaneous, enterovesical, and enterourinary fistulas, except perianal fistulas. Patients who underwent operation for the treatment of perianal disease and strictureplasty were excluded from the analysis. The diagnostic delay interval was categorized as follows: <3 months, 3–6 months, 6–18 months, and ≥18 months.

### Statistical analysis

We first performed two sets of univariate and multivariate analyses to identify the clinical factors associated with the long diagnostic delay in CD patients. In these analyses, the dependent variable was diagnostic delay and the independent variables were as follows: age (<40 or ≥40 years), gender, family history of IBD, any ileal involvement, concomitant upper gastrointestinal (UGI) disease, and disease behavior at the time of diagnosis. The chi-square or Fisher’s exact test was used for the univariate analysis and a multiple logistic regression model adjusted for the aforementioned variables was used for the multivariate analysis.

Further, a Kaplan-Meier survival model and log-rank test were used to evaluate the clinical variables associated with the outcomes of interest (intestinal stenosis, internal fistula, perianal fistula, abscess formation, bowel perforation, and CD-related abdominal surgery). In addition, a multivariate Cox proportional hazards regression analysis including age, gender, family history of IBD, any ileal involvement, UGI involvement, and the diagnostic delay interval was performed to assess the impact of diagnostic delay on the disease course. Hazard ratios (HRs) and 95% confidence intervals (CIs) were calculated as measures of the correlation between the clinical variables and outcomes of interest. Values of *p* < 0.05 were considered statistically significant. Statistical analyses were performed using SPSS software version 18.0 (SPSS Inc., Chicago, IL, USA).

## Results

### Baseline characteristics of patients

This study included 1,047 Korean CD patients. The mean age at first diagnosis was 27.7 ± 12.3 years, and the male-to-female ratio was 2.61:1. Regarding disease location at diagnosis, ileocolonic disease (613 patients, 58.5%) was the most common, and UGI involvement was observed in 95 (9.1%) patients. Disease behavior was inflammatory in 855 (81.7%), stricturing in 104 (9.9%), and penetrating in 88 (8.4%) patients. Of the patients, 748 (71.4%) were on immunomodulators and 304 (29.0%) were prescribed anti-TNFα agents. During the follow-up period, 228 (21.8%) patients underwent abdominal surgery owing to CD-related problems. The mean length of the diagnostic delay was 16.0 ± 33.1 (range: 0–412.4) months. On the basis of the diagnostic delay interval, 471 (45.0%) patients were categorized in <3-month delay group, whereas 224 (21.4%) patients were categorized in ≥18-month delay group. The mean follow-up duration was 89.5 ± 36.1 (range: 6.0–173.3) months. The baseline demographic and clinical features of the study population are outlined in **Table 1**.

**Table 1:** Baseline demographics of study subjects (n = 1,047).

Demographic characteristics		
Age at diagnosis (years)^a^		27.7 ± 12.3 (7.8–87.8)
Gender (male:female)		2.61:1 (757:290)
Family history of IBD (%)		23 (2.2)
History of prior abdominal surgery (%)		154 (14.7)
Disease location at diagnosis (%)^b^		
	Ileum only (L1)	239 (22.8)
	Colon only (L2)	195 (18.6)
	Ileocolon (L3)	613 (58.5)
	Concomitant UGI disease (L4)	95 (9.1)
Disease behavior at diagnosis (%)^b^		
	Inflammatory (B1)	855 (81.7)
	Stricturing (B2)	104 (9.9)
	Penetrating (B3)	88 (8.4)
	Concomitant perianal disease (P)	292 (27.9)
Medication (%)		
	5-ASA	1,025 (97.9)
	Antibiotics	588 (56.2)
	Oral corticosteroids	656 (62.7)
	AZA/6-MP	748 (71.4)
	MTX	12 (1.1)
	Anti-TNFα	304 (29.0)
CD-related abdominal surgery (%)		228 (21.8)
Interval of diagnostic delay (%)		
	< 3 months	471 (45.0)
	3–6 months	151 (14.4)
	6–18 months	201 (19.2)
	≥ 18 months	224 (21.4)
Follow-up duration (months)^a^		89.5 ± 36.1 (6.0–173.3)

IBD, inflammatory bowel disease; UGI, upper gastrointestinal; 5-ASA, 5-aminosalicylic acid; AZA/6-MP, azathioprine/6-mercaptopurine; MTX, methotrexate; anti-TNFα, anti-tumor necrosis factor α; CD, Crohn’s disease.

^a^Mean ± standard deviation.

^b^Disease location and behavior were determined according to the Montreal classification.

### Clinical factors associated with long diagnostic delay in CD patients

We performed univariate and multivariate analyses to evaluate the association between the clinical factors and long diagnostic delay. In CD patients with a diagnostic delay interval of <18 months, compared to those with a diagnostic delay interval of ≥18 months, univariate analysis showed that older age at diagnosis (*p* = 0.006), concomitant UGI involvement (*p* = 0.011), and disease behavior at diagnosis (*p* = 0.004) were associated with long diagnostic delay. Subsequently, we performed a multivariate logistic regression analysis including all clinically significant variables. This analysis identified the following variables as the independent risk factors for long diagnostic delay: older age at diagnosis (≥40 years) (odds ratio [OR] = 1.64; 95% CI, 1.11–2.42, *p* = 0.014), UGI disease (OR = 1.83; 95% CI, 1.14–2.93, *p* = 0.012), and penetrating disease behavior (OR = 2.22; 95% CI, 1.38–3.57, *p* = 0.001) (**Table 2**).

**Table 2:** Clinical factors associated with long diagnostic delay (≥ 18 months) in Crohn’s disease.

		Univariate analysis (Chi-square test)			Multivariate analysis (Logistic regression analysis)		
		Diagnostic delay ≥ 18 months (N = 283)	Diagnostic delay < 18 months (N = 978)	*P* value	OR	95% CI	*P* value
Age at diagnosis (%)							
	≥ 40 years	46 (29.7)	109 (70.3)	0.006	1.64	1.11–2.42	0.014
	< 40 years	178 (20.0)	714 (80.0)		1 (reference)		
Gender							
	Female	72 (24.8)	218 (75.2)	0.094	1.30	0.94–1.81	0.114
	Male	152 (20.1)	605 (79.9)		1 (reference)		
Family history of IBD (%)							
	Yes	8 (34.8)	15 (65.2)	0.113	1.99	0.82–4.87	0.130
	No	216 (21.1)	808 (78.9)		1 (reference)		
Disease location at diagnosis (%)^a^							
	Any ileal involvement	180 (21.1)	672 (78.9)	0.659	0.87	0.59–1.27	0.462
	No involvement of ileum	44 (22.6)	151 (77.4)		1 (reference)		
Concomitant UGI disease (L4)							
	Yes	30 (31.6)	65 (68.4)	0.011	1.83	1.14–2.93	0.012
	No	194 (20.4)	758 (79.6)		1 (reference)		
Disease behavior at diagnosis (%)^a^							
	Inflammatory (B1)	170 (19.9)	685 (80.1)	0.004	1 (reference)		
	Stricturing (B2)	23 (22.1)	81 (77.9)		1.03	0.62–1.70	0.917
	Penetrating (B3)	31 (35.2)	57 (64.8)		2.22	1.38–3.57	0.001

OR, Odds ratio; CI, confidence interval; IBD, inflammatory bowel disease; UGI, upper gastrointestinal.

^a^Disease location and behavior were determined according to the Montreal classification.

### Diagnostic delay was a predictive risk factor for intestinal complications in CD patients

During longitudinal follow-up, disease-related complications developed in some CD patients in this study. Intestinal stenosis, internal fistula, perianal fistula, abscess formation, and intestinal perforation occurred in 262 (25.0%), 168 (16.0%), 390 (37.2%), 133 (12.7%), and 50 (4.8%) patients, respectively. To evaluate the impact of diagnostic delay on the clinical course in CD patients, associations between clinical factors including the diagnostic delay interval and the development of these complications were analyzed using univariate (Kaplan-Meier survival model) and multivariate (Cox proportional hazards regression model) models.

In this study, long diagnostic delay (≥18 months) was independently associated with an increased risk of intestinal stenosis (HR = 1.43; 95% CI, 1.07–1.93, *p* = 0.017). Moreover, a significant association was observed between ileal involvement and the risk of intestinal stenosis (HR = 1.54; 95% CI, 1.08–2.21, *p* = 0.017) (**Table 3**). Similarly, diagnostic delay and ileal involvement were significantly associated with an increased risk of internal fistulas (HR = 1.62; 95% CI, 1.12–2.33, *p* = 0.011 and HR = 1.89; 95% CI, 1.17–3.06, *p* = 0.009, respectively) (**Table 4**). In addition, long diagnostic delay (≥18 months) was independently predictive of further development of perianal fistulas (HR = 1.38; 95% CI, 1.06–1.80, *p* = 0.016) (**Table 5**).

**Table 3:** Predictive clinical factors associated with the risk of intestinal stenosis in Korean patients with Crohn’s disease.

		Univariate analysis^a^		Multivariate analysis^b^		
		5-year cumulative rate (%)	*P* value	HR	95% CI	*P* value
Age at diagnosis (%)			0.164			
	< 40 years	21.3		0.81	0.58–1.13	0.211
	≥ 40 years	27.5		1 (Ref)		
Gender			0.176			
	Male	23.5		1.25	0.94–1.66	0.121
	Female	19.1		1 (Ref)		
Family history of IBD (%)			0.136			
	Yes	31.2		1.46	0.75–2.84	0.272
	No	22.0		1 (Ref)		
Disease location at diagnosis (%)^c^			0.013			
	Any ileal involvement	23.3		1.54	1.08–2.21	0.017
	No involvement of ileum	17.5		1 (Ref)		
Concomitant UGI disease (L4)			0.095			
	Yes	30.0		1.26	0.86–1.84	0.242
	No	21.5		1 (Ref)		
Interval of diagnostic delay (%)			0.017			
	< 3 months	21.2		1 (Ref)		
	3–6 months	19.2		0.87	0.58–1.29	0.477
	6–18 months	21.0		0.97	0.69–1.36	0.846
	≥ 18 months	27.7		1.43	1.07–1.93	0.017

HR, hazard ratio; CI, confidence interval; IBD, inflammatory bowel disease; UGI, upper gastrointestinal.

^a^calculated by a Kaplan-Meier survival model.

^b^analyzed by a multivariate Cox proportional hazards regression model.

^c^Disease location and behavior were determined according to the Montreal classification.

**Table 4:** Predictive clinical factors associated with the risk of internal fistula in Korean patients with Crohn’s disease.

		Univariate analysis^a^		Multivariate analysis^b^		
		5-year cumulative rate (%)	*P* value	HR	95% CI	*P* value
Age at diagnosis (%)			0.354			
	< 40 years	13.2		1.34	0.84–2.15	0.221
	≥ 40 years	12.7		1 (Ref)		
Gender			0.211			
	Male	12.5		0.81	0.59–1.12	0.207
	Female	14.9		1 (Ref)		
Family history of IBD (%)			0.893			
	Yes	8.7		0.89	0.33–2.41	0.818
	No	13.3		1 (Ref)		
Disease location at diagnosis (%)^c^			0.009			
	Any ileal involvement	14.2		1.89	1.17–3.06	0.009
	No involvement of ileum	8.5		1 (Ref)		
Concomitant UGI disease (L4)			0.829			
	Yes	14.2		0.99	0.59–1.66	0.958
	No	13.1		1 (Ref)		
Interval of diagnostic delay (%)			0.040			
	< 3 months	11.7		1 (Ref)		
	3–6 months	12.5		1.01	0.63–1.64	0.957
	6–18 months	10.2		0.92	0.59–1.43	0.720
	≥ 18 months	19.4		1.62	1.12–2.33	0.011

HR, hazard ratio; CI, confidence interval; IBD, inflammatory bowel disease; UGI, upper gastrointestinal.

^a^calculated by a Kaplan-Meier survival model.

^b^analyzed by a multivariate Cox proportional hazards regression model.

^c^Disease location and behavior were determined according to the Montreal classification.

**Table 5:** Predictive clinical factors associated with the risk of perianal fistula in Korean patients with Crohn’s disease.

		Univariate analysis^a^		Multivariate analysis^b^		
		5-year cumulative rate (%)	*P* value	HR	95% CI	*P* value
Age at diagnosis (%)			< 0.001			
	< 40 years	39.1		3.15	2.05–4.86	< 0.001
	≥ 40 years	14.5		1 (Ref)		
Gender			0.152			
	Male	37.0		1.12	0.89–1.41	0.339
	Female	31.3		1 (Ref)		
Family history of IBD (%)			0.136			
	Yes	47.8		1.46	0.82–2.61	0.198
	No	35.1		1 (Ref)		
Disease location at diagnosis (%)^c^			0.557			
	Any ileal involvement	35.8		1.03	0.80–1.34	0.808
	No involvement of ileum	33.9		1 (Ref)		
Concomitant UGI disease (L4)			0.359			
	Yes	40.1		1.12	0.80–1.56	0.523
	No	35.0		1 (Ref)		
Interval of diagnostic delay (%)			0.005			
	< 3 months	29.9		1 (Ref)		
	3–6 months	40.4		1.42	1.06–1.90	0.020
	6–18 months	40.7		1.42	1.09–1.85	0.009
	≥ 18 months	39.0		1.38	1.06–1.80	0.016

HR, hazard ratio; CI, confidence interval; IBD, inflammatory bowel disease; UGI, upper gastrointestinal.

^a^calculated by a Kaplan-Meier survival model.

^b^analyzed by a multivariate Cox proportional hazards regression model.

^c^Disease location and behavior were determined according to the Montreal classification.

However, no significant association was observed between long diagnostic delay and other complications such as abscess formation and intestinal perforation. Multivariate analysis showed that no clinical factors were associated with a risk of abscess formation (**S1 Table**). A significant association was observed between intestinal perforation and the male gender (HR = 2.16, *p* = 0.047), but not between intestinal perforation and diagnostic delay (**S2 Table**). As for the first CD-related abdominal surgery, no significant association was observed between long diagnostic delay and the risk of abdominal surgery. Other factors such as ileal involvement (HR = 1.66, *p* = 0.014), stricturing (HR = 4.83, *p* < 0.001), and penetrating behavior (HR = 5.99, *p* < 0.001) at diagnosis were associated with abdominal surgery (**S3 Table**).

## Discussion

This nationwide CD cohort study in Korea showed that long diagnostic delay was significantly associated with an increased risk of CD-related complications, such as intestinal stenosis, internal fistulas, and perianal fistulas. Up to date, few studies have evaluated the impact of the diagnostic delay of CD. A Swiss IBD cohort study reported that a long diagnostic delay was associated with the further development of bowel stenosis and intestinal surgery [18]. These results may be closely related to the sustained changes in the disease behavior of CD. Inflammatory disease behavior is dominant during the early stages of CD, whereas patients tend to develop intestinal complications such as strictures or fistulas as time passes [1,2]. In a study by Louis et al., disease behavior changed in 45.9% of the CD patients over 10 years, and the change from inflammatory disease to either stricturing or penetrating disease was the most common (27.1% and 29.4%, respectively) [2]. Accordingly, it is speculated that a long diagnostic delay can represent a treatment gap that causes disease progression to intestinal complications. Therefore, the diagnostic delay of CD is an important issue because stricturing and penetrating disease behavior are both independent risk factors for an unfavorable disease course, such as CD-related abdominal surgery [19,20].

In addition, in the last two decades, several studies reported that an early intensive treatment with immunosuppressive agents or biologics showed better clinical outcomes than conventional treatment in newly diagnosed CD patients [3,4]. In this regard, an international group of IBD experts suggested that the concept of “early CD” be used in future clinical trials of CD [21]. They defined early CD as a disease duration of ≤18 months measured from the first diagnosis, with no current/previous treatment with immunomodulators or biologics. Regarding the early use of disease-modifying agents, a trial, in which 133 moderate-severe CD patients were randomized to receive either conventional treatment (step-up approach) or early combined treatment with azathioprine and infliximab (top-down approach), reported that the combined immunosuppression group (top-down) showed a greater effectiveness in clinical remission than the step-up group at week 26 (60.0% vs. 35.9%, *p* = 0.0062) [3]. The impact of disease duration on treatment response was also suggested in the PRECISE 2 study. This study reported that at week 26, the clinical response rate with certolizumab pegol in CD patients with a diagnosis < 1 year (89.5%) was better than that in those with a diagnosis ≥ 5 years (57.3%, *p* < 0.05) [4]. Furthermore, mucosal healing, which is a promising surrogate end point for estimating disease course early, was achieved more frequently in CD patients receiving scheduled infliximab treatment than in those receiving episodic treatment for CD [22]. Taken together, the earlier CD was diagnosed from the first CD-related symptoms, the earlier intensive treatment with disease-modifying drugs was given to more CD patients, and the better was the disease course.

Interestingly, in this study, the impact of long diagnostic delay was observed on the occurrence of intestinal stenosis and internal fistula among the CD-related intestinal complications. This phenomenon can be explained by the significant association between the occurrence of intestinal stenosis and internal fistulas. Although the exact pathogenesis of developing an internal fistula is still unclear, a previous study showed that most internal fistulas occur within or close (at the proximal end) to intestinal stenosis [23]. In addition, a significant association was observed between a long diagnostic delay and occurrence of a perianal fistula. The occurrence of a perianal fistula is a clinical predictive factor for disabling disease and disease-related surgery [24–26] as well as a clinical outcome of an unfavorable disease course. These factors may cause this significant association. On the other hand, there was no significant association between long diagnostic delay and the risk of abdominal surgery. These findings can be explained by several factors. First, not all intestinal stenosis or fistulas require surgery. One researcher reported that the intestinal fistula in itself is not regarded an indication for surgery, although it frequently occurs in CD patients [27]. In general, fistulas accompanied by intestinal obstructions or abscesses, or which cause intestinal malabsorption or communicate with the genitourinary tract are indications for surgery. As for intestinal stenosis, abdominal surgery is only one of the therapeutic options. The other options are anti-inflammatory medication and endoscopic balloon dilation [28,29]. Endoscopic dilation is an effective and safe alternative to surgical management, especially in short stenosis [29]. Additionally, the present study did not include strictureplasty, a surgery used for intestinal stenosis. Second, the cumulative rate of first CD-related abdominal surgery in Korean CD patients was much lower than that in Western patients [13,30]. The cumulative operation rate could be influenced by differences of ethnicity, environment, or attitudes of physicians and patients to surgery. Korean patients might be more unwilling to undergo intestinal resection than Westerners, possibly because Korea is a country based on Confucian values [30,31]. These factors can partially explain why we found no significant association between long diagnostic delay and the risk of abdominal surgery in this study.

Our study also evaluated the clinical factors associated with a long diagnostic delay in CD patients. Consequently, older age at diagnosis (≥40 years), concomitant UGI disease, and penetrating disease behavior were identified as independent risk factors for long diagnostic delay (≥18 months). Among them, an increased risk of diagnostic delay in older CD patients might mainly depend on physician’s delay in diagnosing CD. Considering that the usual onset of CD is at a younger age and there is still a relatively low prevalence of CD in Korea [13,32], it is speculated that physicians tend to not list CD in their differential diagnosis when they meet older patients with CD symptoms. The increased risk of diagnostic delay in patients with concomitant UGI disease may be partially explained by the fact that clinical symptoms of CD are similar to those with functional gastrointestinal disorders [33] or other UGI diseases including peptic ulcer or dyspepsia. In particular, peptic ulcer is a significant concern for Koreans, in whom the prevalence of *Helicobacter pylori* (*H*. *pylori*) infection for individuals older than 16 years is 59.6% [34]. *H*. *pylori* has been reported to play a causal role in various conditions such as peptic ulcer disease [35], gastroesophageal reflux disease [36], and functional dyspepsia [37]. Additionally, UGI disease in CD, namely, the Montreal L4 category also includes the involvement in the proximal part of the small bowel as well as esophageal and gastroduodenal involvement [15,38]. The diagnosis of small bowel CD, especially proximal small bowel, may be challenging because of various nonspecific symptoms and limitations of detection with conventional examinations [39]. Regarding the association between disease behavior and diagnostic delay, the penetrating behavior may be considered to be a consequence of long diagnostic delay. CD patients with long diagnostic delay may tend to develop penetrating disease behavior because of the sustained progression of the disease behavior over time [1,2]. As for this issue, a few studies have evaluated the factors associated with the diagnostic delay of CD. In a Swiss IBD cohort study by Vavricka et al., younger age at first diagnosis (<40 years) and ileal involvement were significantly associated with a long diagnostic delay (≥24 months) [8]. A French cohort study reported that no medical and socioeconomic characteristics affected the long diagnostic delay of CD [9]. A recent study in Chinese CD patients indicated older age at diagnosis (> 40 years), a basic education status, and no family history of CD were risk factors for a long diagnostic delay [10]. The results of these studies are not comparable to our results. This discrepancy may be influenced by differences in the epidemiologic and clinical features of CD and genetic susceptibility to CD according to patient ethnicity [13,30,32,40,41]. Further large studies across multiple ethnic groups are warranted to obtain convincing results related to clinical factors associated with a long diagnostic delay in CD patients.

Our study has several limitations. First, because of the retrospective design of this study, it may be subject to recall bias for the first CD-related symptoms and their onset. In addition, since some symptoms of CD are similar to those of IBS, it was difficult to determine the exact duration of the diagnostic delay (from the first symptoms to diagnosis) in some cases. Additionally, available data retrieved from the CONNECT study, a multicenter retrospective cohort, did not include the detailed information, such as the different diagnostic intervals (patient-related or physician-related delay) or how many doctors they had visited. Thus, in the present study, we assessed and analyzed clinical data including only the “overall diagnostic delay” (the time interval from the onset of the first symptoms to the establishment of the CD diagnosis). Second, our study lacks some important information on environmental and socioeconomic factors such as smoking habits, intake of non-steroidal anti-inflammatory drugs or oral contraceptives, and socioeconomic or educational status of the patients. In particular, previous studies have determined the harmful effects of smoking habits on disease course and the risk of recurrence or reoperation in CD patients [42–44]. Moreover, this study was not a population-based cohort study. However, considering that the majority of Korean CD patients are diagnosed and managed in tertiary medical centers in Korea [32], this CD cohort, including patients from 32 hospitals nationwide, may considerably reflect the real population of CD patients in Korea. Furthermore, since all patients were diagnosed and treated by IBD specialists, clinical data in this cohort are quite convincing.

In conclusion, this study demonstrated that a long diagnostic delay is significantly associated with an increased risk of CD-related complications such as intestinal stenosis, internal fistulas, and perianal fistulas. Moreover, older age at diagnosis (≥40 years), concomitant UGI involvement, and penetrating disease behavior are closely associated with diagnostic delay in CD patients. On the basis of these results, reducing the diagnostic delay of CD may reduce the occurrence of many disabling complications associated with CD. Thus, our results suggest that physicians should make particular efforts to increase the rate of early diagnosis and raise public awareness about the warning symptoms of CD.

## Supporting Information

S1 Table Predictive clinical factors associated with the risk of abscess formation in Korean patients with Crohn’s disease. (DOC)

S2 Table Predictive clinical factors associated with the risk of intestinal perforation in Korean patients with Crohn’s disease. (DOC)

S3 Table Predictive clinical factors associated with first disease-related abdominal surgery in Korean patients with Crohn’s disease. (DOC)
