# Supplementary material for: Clinical Factors and Disease Course Related to Diagnostic Delay in Korean Crohn’s Disease Patients: Results from the CONNECT Study
Source: PLoS One. 2015 Dec 8;10(12):e0144390. doi: 10.1371/journal.pone.0144390 (PMC4672933; doi:10.1371/journal.pone.0144390)
Supplement: S1 Table — (DOC) [file pone.0144390.s001.doc]

**Supporting Table 1.** Predictive clinical factors associated with the risk of abscess formation in Korean patients with Crohn’s disease

|  | Univariate analysis^†^ | |  | Multivariate analysis^‡^ | | |
| --- | --- | --- | --- | --- | --- | --- |
|  | 5-year cumulative rate (%) | *P* value |  | HR | 95% CI | *P* value |
| Age at diagnosis (%) |  | 0.125 |  |  |  |  |
| < 40 years | 11.2 |  |  | 1.58 | 0.89 – 2.81 | 0.120 |
| ≥ 40 years | 8.5 |  |  | 1 (Ref) |  |  |
| Gender |  | 0.814 |  |  |  |  |
| Male | 10.9 |  |  | 1.01 | 0.69 – 1.48 | 0.973 |
| Female | 10.6 |  |  | 1 (Ref) |  |  |
| Family history of IBD (%) |  | 0.942 |  |  |  |  |
| Yes | 13.3 |  |  | 0.97 | 0.31 – 3.06 | 0.961 |
| No | 10.8 |  |  | 1 (Ref) |  |  |
| Disease location at diagnosis (%)^§^ |  | 0.362 |  |  |  |  |
| Any ileal involvement | 11.1 |  |  | 1.22 | 0.76 – 1.94 | 0.415 |
| No involvement of ileum | 9.7 |  |  | 1 (Ref) |  |  |
| Concomitant UGI disease (L4) |  | 0.046 |  |  |  |  |
| Yes | 16.0 |  |  | 1.58 | 0.95 – 2.60 | 0.076 |
| No | 10.3 |  |  | 1 (Ref) |  |  |
| Interval of diagnostic delay (%) |  | 0.341 |  |  |  |  |
| < 3 months | 12.2 |  |  | 1 (Ref) |  |  |
| 3–6 months | 7.7 |  |  | 0.59 | 0.32 – 1.09 | 0.090 |
| 6–18 months | 10.9 |  |  | 0.88 | 0.56 – 1.40 | 0.600 |
| ≥ 18 months | 10.0 |  |  | 1.03 | 0.67 – 1.58 | 0.896 |

HR, hazard ratio; CI, confidence interval; IBD, inflammatory bowel disease; UGI, upper gastrointestinal.

^†^calculated by a Kaplan-Meier survival model

^‡^analyzed by a multivariate Cox proportional hazards regression model

^§^Disease location and behavior were determined according to the Montreal classification.
