# Supplementary material for: Clinical Factors and Disease Course Related to Diagnostic Delay in Korean Crohn’s Disease Patients: Results from the CONNECT Study
Source: PLoS One. 2015 Dec 8;10(12):e0144390. doi: 10.1371/journal.pone.0144390 (PMC4672933; doi:10.1371/journal.pone.0144390)
Supplement: S2 Table — (DOC) [file pone.0144390.s002.doc]

**Supporting Table 2.** Predictive clinical factors associated with the risk of intestinal perforation in Korean patients with Crohn’s disease

|  | Univariate analysis^†^ | |  | Multivariate analysis^‡^ | | |
| --- | --- | --- | --- | --- | --- | --- |
|  | 5-year cumulative rate (%) | *P* value |  | HR | 95% CI | *P* value |
| Age at diagnosis (%) |  | 0.126 |  |  |  |  |
| < 40 years | 3.9 |  |  | 0.55 | 0.28 – 1.09 | 0.085 |
| ≥ 40 years | 7.4 |  |  | 1 (Ref) |  |  |
| Gender |  | 0.054 |  |  |  |  |
| Male | 5.1 |  |  | 2.16 | 1.01 – 4.62 | 0.047 |
| Female | 2.5 |  |  | 1 (Ref) |  |  |
| Family history of IBD (%)^§^ |  |  |  | not included |  |  |
| Yes | - | - |  |  |  |  |
| No | - |  |  |  |  |  |
| Disease location at diagnosis (%)^¶^ |  | 0.218 |  |  |  |  |
| Any ileal involvement | 4.8 |  |  | 1.71 | 0.73 – 4.01 | 0.220 |
| No involvement of ileum | 2.7 |  |  | 1 (Ref) |  |  |
| Concomitant UGI disease (L4) |  | 0.823 |  |  |  |  |
| Yes | 5.5 |  |  | 1.05 | 0.41 – 2.64 | 0.926 |
| No | 4.3 |  |  | 1 (Ref) |  |  |
| Interval of diagnostic delay (%) |  | 0.793 |  |  |  |  |
| < 3 months | 4.9 |  |  | 1 (Ref) |  |  |
| 3–6 months | 2.0 |  |  | 0.64 | 0.24 – 1.66 | 0.357 |
| 6–18 months | 4.7 |  |  | 0.85 | 0.40 – 1.83 | 0.683 |
| ≥ 18 months | 4.5 |  |  | 0.90 | 0.44 – 1.83 | 0.761 |

HR, hazard ratio; CI, confidence interval; IBD, inflammatory bowel disease; UGI, upper gastrointestinal.

^†^calculated by a Kaplan-Meier survival model

^‡^analyzed by a multivariate Cox proportional hazards regression model

^§^Statistical value can not be measured because there is no event in one group (family history of IBD). Thus, this variable (family history of IBD) was not included in multivariate analysis.

^¶^Disease location and behavior were determined according to the Montreal classification.
