# Supplementary material for: Clinical Factors and Disease Course Related to Diagnostic Delay in Korean Crohn’s Disease Patients: Results from the CONNECT Study
Source: PLoS One. 2015 Dec 8;10(12):e0144390. doi: 10.1371/journal.pone.0144390 (PMC4672933; doi:10.1371/journal.pone.0144390)
Supplement: S3 Table — (DOC) [file pone.0144390.s003.doc]

**Supporting Table 3.** Predictive clinical factors associated with first disease-related abdominal surgery in Korean patients with Crohn’s disease

|  | Univariate analysis^†^ | |  | Multivariate analysis^‡^ | | |
| --- | --- | --- | --- | --- | --- | --- |
|  | 5-year cumulative rate (%) | *P* value |  | HR | 95% CI | *P* value |
| Age at diagnosis (%) |  |  |  |  |  |  |
| < 40 years | 17.7 | 0.022 |  | 0.82 | 0.58 – 1.15 | 0.246 |
| ≥ 40 years | 27.3 |  |  | 1 (Ref) |  |  |
| Gender |  | 0.715 |  |  |  |  |
| Male | 19.3 |  |  | 1.02 | 0.76 – 1.37 | 0.888 |
| Female | 18.9 |  |  | 1 (Ref) |  |  |
| Family history of IBD (%) |  | 0.864 |  |  |  |  |
| Yes | 13.5 |  |  | 0.86 | 0.35 – 2.10 | 0.740 |
| No | 19.3 |  |  | 1 (Ref) |  |  |
| Disease location at diagnosis (%)^§^ |  | 0.003 |  |  |  |  |
| Any ileal involvement | 20.9 |  |  | 1.66 | 1.11 – 2.49 | 0.014 |
| No involvement of ileum | 11.7 |  |  | 1 (Ref) |  |  |
| Concomitant UGI disease (L4) |  | 0.364 |  |  |  |  |
| Yes | 22.5 |  |  | 0.97 | 0.63 – 1.49 | 0.879 |
| No | 18.8 |  |  | 1 (Ref) |  |  |
| Disease behavior at diagnosis (%)^§^ |  | < 0.001 |  |  |  |  |
| Inflammatory (B1) | 11.7 |  |  | 1 (Ref) |  |  |
| Stricturing (B2) | 48.9 |  |  | 4.83 | 3.48 – 6.72 | < 0.001 |
| Penetrating (B3) | 56.3 |  |  | 5.99 | 4.28 – 8.38 | < 0.001 |
| Interval of diagnostic delay (%) |  | 0.073 |  |  |  |  |
| < 3 months | 20.9 |  |  | 1 (Ref) |  |  |
| 3–6 months | 12.6 |  |  | 0.64 | 0.41 – 1.02 | 0.059 |
| 6–18 months | 17.5 |  |  | 0.84 | 0.58 – 1.21 | 0.344 |
| ≥ 18 months | 21.4 |  |  | 0.87 | 0.63 – 1.21 | 0.402 |

HR, hazard ratio; CI, confidence interval; IBD, inflammatory bowel disease; UGI, upper gastrointestinal.

^†^calculated by a Kaplan-Meier survival model

^‡^analyzed by a multivariate Cox proportional hazards regression model

^§^Disease location and behavior were determined according to the Montreal classification.
